# Truncal valve management: The keystone of success

**DOI:** 10.1016/j.xjon.2023.06.008

**Published:** 2023-07-01

**Authors:** Phillip S. Naimo, Igor E. Konstantinov

**Affiliations:** a Department of Cardiothoracic Surgery, Royal Children's Hospital, Melbourne, Victoria, Australia; b Department of Paediatrics, University of Melbourne, Melbourne, Victoria, Australia; c Heart Research Group, Murdoch Children's Research Institute, Melbourne, Victoria, Australia; d Melbourne Children's Centre for Cardiovascular Genomics and Regenerative Medicine, Melbourne, Victoria, Australia

To the Editor:

**Figure:**
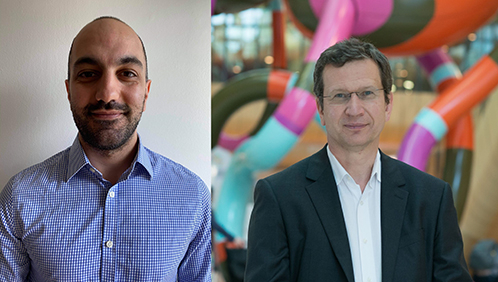

The authors reported no conflicts of interest.

The *Journal* policy requires editors and reviewers to disclose conflicts of interest and to decline handling or reviewing manuscripts for which they may have a conflict of interest. The editors and reviewers of this article have no conflicts of interest.

We read with interest the recent article by Hoashi and colleagues^1^ reporting their experience with truncus arteriosus repair in 50 patients between 1978 and 2020. This study of 50 patients with truncus arteriosus, spanning over 4 decades, demonstrated an overall survival of 68.8% at 30 years, with mean follow-up of 15 years. This overall survival is not atypical for a study spanning such a large time period, particularly given that earlier procedures involved staged, palliative surgery as the index operation. Hoashi and colleagues^1^ performed cardiopulmonary exercise testing in at a median time of 19.7 years after truncus repair, which showed mildly reduced exercise capacity. They provided novel insights to a correlation between a dilated truncal root and reduced exercise tolerance, although there are a number of other factors that may be at play, including, but not limited to, right and/or left ventricular dysfunction and significant pulmonary or tricuspid regurgitation.

Amongst the findings, Hoashi and colleagues^1^ identified truncal valve regurgitation as risk factors for both survival and reoperation. Patients in this study underwent either bicuspidization or tricuspidization with commissure closure and plication of the regurgitant intercommissural space. It is unclear how many patients underwent truncal valve repair at the initial operation and have many at a later reoperation. We have previously shown that mild truncal valve regurgitation is well tolerated^2^; however, moderate or severe truncal valve regurgitation, particularly in the quadricuspid truncal valve, often require surgical intervention.^3^^,^^4^ In these cases, truncal valve repair is not only achievable, but also durable if annular reduction is undertaken.^2^, ^3^, ^4^, ^5^ We have demonstrated excellent results with cusp resection and annular reduction, with an overall survival in these patients of 77% at 15 years and freedom from truncal valve reoperation of 64% at 10 years. Annular reduction is crucial to success. The annular reduction makes truncal valve repair, first and foremost, durable, and, as such, avoids the need for truncal valve replacement with a mechanical prosthesis which would require lifelong anticoagulation.

We agree with the authors’ statement that the key to survival in patients with truncus arteriosus is the management of the truncal valve. Over time, we have seen improvements and innovations in the perioperative management and surgical techniques that have mitigated the traditional risk factors of low birth weight and aortic arch obstruction. In the current era, the keystone to successful truncus arteriosus repair is addressing the regurgitant truncal valve.
